# A New Parameter of Hip Instability in Developmental Dysplasia of the Hip (DDH): Teardrop Distance

**DOI:** 10.3389/fsurg.2022.899960

**Published:** 2022-06-14

**Authors:** Guoyue Yang, Zhendong Zhang, Xiaobin Hou, Dianzhong Luo, Hui Cheng, Kai Xiao, Hui Liu, Hong Zhang

**Affiliations:** ^1^ Department of Orthopedics, The Third Central Hospital of Tianjin, Tianjin, China; ^2^ Tianjin Institute of Hepatobiliary Disease, Tianjin, China; ^3^ Tianjin Key Laboratory of Artificial Cell, Tianjin, China; ^4^ Artificial Cell Engineering Technology Research Center of Public Health Ministry, Tianjin, China; ^5^ Department of Orthopedics, The Fourth Medical Center of the Chinese People’s Liberation Army General Hospital, Beijing, China

**Keywords:** hip instability, developmental dysplasia of the hip, osteotomy, AP pelvic radiograph, teardrop distance

## Abstract

**Background:**

Hip instability is one of the etiologies of accelerated onset of osteoarthritis in developmental dysplasia of the hip (DDH). There are some radiological parameters for hip instability in hip dysplasia like broken shenton’s line, elevated acetabular index, reduced lateral center edge angle (LCEA), upsloping lateral sourcil. We have discovered a new index of teardrop distance (TD) for assessing instability. Herein, we hypothesized that increased TD could be used as evidence of hip instability in DDH patients, which we verified using TD as an auxiliary diagnostic parameter for DDH, from supine to standing position.

**Methods:**

Female DDH patients undergoing Bernese periacetabular osteotomy (PAO) were enrolled in the DDH group, and normal female volunteers were in the control group. Anteroposterior radiographs of the pelvis in the supine and standing positions were taken, and LCEA, Tönnis angle (TA), sharp angle (SA), and TD were tested using Stata software to analyze the changes between supine and standing anteroposterior pelvic radiographs.

**Results:**

There were 26 female volunteers with 52 hips in the control group: supine TD 6.80 ± 0.98 mm, standing TD 6.65 ± 1.3 mm (*P* > 0.05). A total of 78 patients with 135 hips were included in the DDH group: supine TD 10.51 ± 3.50 mm, standing TD 10.93 ± 4.23 mm (*P* < 0.05). In either supine or standing position, TD in the DDH group was significantly wider than that in the control group (*P* < 0.05). In the DDH group, TD was correlated with TA and LCEA (rp 0.494–0.588, *P* < 0.05); TD was not correlated with SA, weight, or BMI (*P* > 0.05). There was a weak correlation between TD difference and standing LCEA (rp −0.276, *P* < 0.05).

**Conclusion:**

TD > 10 mm was a common imaging feature of DDH. It increased from supine to standing position, thus indicating hip instability in DDH patients. The hip parameters of both positions should be compared, fully considering the factors of hip stability.

## Introduction

Cooperman et al. reported the concept of hip instability and its role in the degenerative process of the joint (1). Poor bony coverage of the acetabulum, femoroacetabular impingement (FAI), and soft tissue relaxation may be the basis for clinical diagnosis of hip instability (2–5). Multiple factors contribute to hip instability in adolescents and young adults, and DDH is a common pathological mechanism of hip instability. A flattening of the acetabulum dome or a greater lateral inclination may indicate poor femoral head coverage and poor match between the acetabulum and femoral head, thus leading to hip instability (6–8). On the premise of poor femoral head coverage, hip instability accelerates the occurrence of osteoarthritis. Therefore, one of the key factors in osteoarthritis secondary to DDH is the unstable state of the hip joint. Hip preservation surgeries aim to obtain a concentric relationship of the femoral head and acetabulum and maintain a stable hip joint (9).

There are some studies that have reported the measurement of in vivo kinematics of the hip. Invasive bone pins with marker clusters can access the motion of the bones directly, but it could restrict body motion and increase the risk of infection. Magnetic resonance imaging and computerized tomography (CT) scan has been used to reconstruct 3D bone models for measurement of the poses of the hip and for determination of the hip center position, but the measurement is restricted to stationary postures. Radio stereometric analysis (RSA) has also been applied to measure the in vivo 3D kinematics of the joint by tracking the position of implanted titanium beads. However, this invasive technique limits the subject population to patients who have undergone surgical intervention and is difficult to be applied to the native hip joint (10–15). Some hip indicators based on anteroposterior pelvic radiographs, such as reduced LCEA, subluxated femoral head, deficient acetabular wall with concomitant insufficient femoral coverage, positive femoro-epiphyseal acetabular roof (FEAR) index, upsloping lateral sourcil, can also indicate hip instability. We have discovered a new index of TD for assessing instability. From the supine position to the standing position, the change of TD can determine whether there is instability of the hip joint in DDH under weight-bearing conditions. The purpose of this study is to verify the reliability of TD as a radiological parameter assessing hip instability in DDH patients.

## Materials and Methods

The study was approved by the Ethics Committee of The Third Central Hospital of Tianjin. Female DDH patients undergoing Bernese periacetabular osteotomy (PAO) were enrolled in the DDH group, and normal female volunteers were in the control group. Anteroposterior X-ray films of the pelvis were taken in the supine position and standing positions. Inclusion criteria of the DDH group: (1) aged 18–50 years, with a chief complaint of hip discomfort or mild pain; (2) no obvious pelvic tilt, and hip mobility was in the normal range; (3) anteroposterior radiographs of the pelvis in the supine and standing positions taken according to standard procedure; (4) LCEA <20°, Hartofilakidis type I (16); (5) osteoarthritis Tönnis grade 0 I, II (17). Exclusion criteria: (1) spinal deformity and history of the hip disease; (2) history of pelvic and lower extremity fractures or surgeries; (3) history of ipsilateral or contralateral hip surgery; (4) over 18 years old without a closed epiphysis.

**Figure 1:**
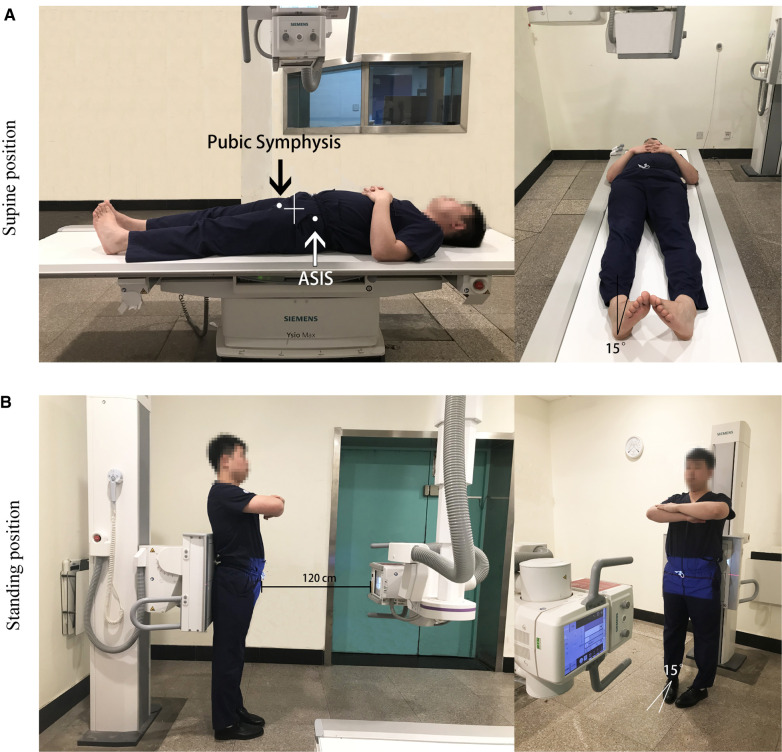
Standard body positions of AP pelvic radiographs. (**A**) The AP pelvic radiographs of supine position. (**B**) The AP pelvic radiographs of standing position. ASIS is anterior superior iliac spine.

The AP pelvic radiographs of supine position require patients to keep the lower limbs parallel, feet within 15°–20° internal rotation, and both the patella point anteriorly. The AP pelvic radiographs of standing position require the patient to stand, both lower limbs parallel and bipedal internal rotation. The tube was placed at the center of the pubic symphysis and the two anterior superior iliac spine. The distance between the tube and film was 120 cm in all cases. The film will be saved in JPG format. Age, gender, height, weight, number in random order were recorded. The radiative magnification was measured by the X-ray of a known length of an object. The relevant parameters were measured using Uniweb Viewer version 4.0 software (Figure 1).

**Figure 2:**
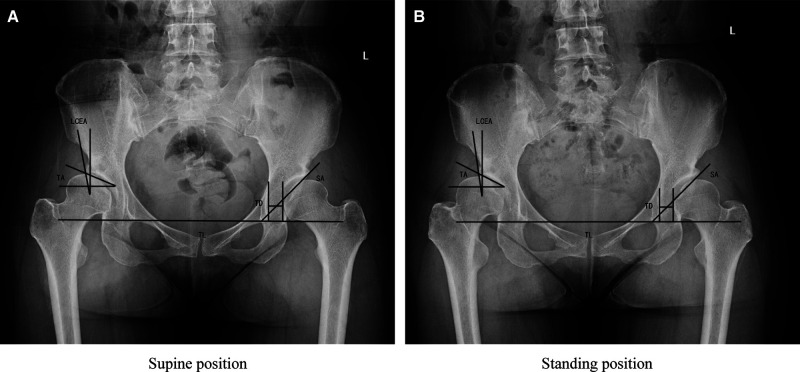
Supine (**A**) and standing (**B**) AP pelvic radiograph are shown. TL is the teardrop line; LCEA and TA are the lateral center edge angle and Tönnis angle, TD and SA are the teardrop distance and sharp angle. TD in the standing position was larger than that in the supine position.

All images were uploaded to a commercially available digital radiographic viewing program (GE Definium 6000DR X-ray machine, Unisight Image Processing System GE, USA).The degree of DDH was assessed by SA, TA, and LCEA, using TD as a parameter reflecting the positional relationship between the acetabulum and the femoral head (17–19). The line of the lower edge of both tears was taken as the horizontal reference line of the pelvis, and the vertical one was used as the vertical reference line (20). TD was the horizontal distance between the lateral edge of the teardrop and the inner edge of the femoral head (Figure 2).

### Statistical Analysis

Stata 9.2 package was used for statistical analysis. Paired t-test was used for the data with a normal distribution when comparing imaging parameters between supine and standing positions. Relationships of TD and other parameters were analyzed by Pearson correlation analysis. *P* < 0.05 was considered statistically significant. Pearson correlation analysis was used for testing intraobserver and interobserver agreement.

## Results

A total of 78 female DDH patients with 135 hips, aged 18–49 years (average of 27.59 years) treated from December 2015 to May 2019 who met the inclusion and exclusion criteria were enrolled. The control group included 26 normal female volunteers with 52 hips, aged 18–42 years, with an average of 25.75 years.

There was no significant difference in TD between the supine position and the standing position in the control group. TD of the DDH group was significantly wider than that of the control group in both supine and standing positions, indicating that TD > 10 mm is the positive imaging finding of DDH in females. In the DDH group, TD in the standing position was larger than that in the supine position, and there was a significant difference. It was indicated that a certain extent of horizontal displacement of the femoral head occurred in DDH patients with weight-bearing, which is a manifestation of hip instability (Table 1).

**Table 1:** TD difference between supine and standing positions.

	*n*	Supine ($\bar{x}\pm s$)	Standing ($\bar{x}\pm s$)	*P*-value
Control group	52	6.80 ± 0.98	6.65 ± 1.30	0.131
DDH group	135	10.50 ± 3.51	10.93 ± 4.23	0.019
*P*-value		<0.0001	<0.0001	

In the DDH group, TD was correlated with TA and LCEA (rp 0.494–0.588, *P* < 0.05); TD and SA were not correlated (*P* > 0.05). It indicated a correlation between TD and the degree of DDH. The more severe dysplastic deformity, the greater TD. TD was not significantly associated with weight or BMI (Table 2). There was a weak correlation between TD changes and standing LCEA (rp-0.276, *P* < 0.05) (Table 3). When TD > 10 mm, there was a weak correlation between TD changes and LCEA in the standing position in 69 hips (rp-0.313 *P* < 0.05) (Table 3).

**Table 2:** Correlation between TD and other hip parameters.

	Supine TD		Standing TD	
	Pearson Correlation	*P*-value	Pearson Correlation	*P*-value
Supine TA	0**.**617	0**.**000	0**.**596	<0**.**001
Standing TA	0**.**587	0**.**000	0**.**580	<0**.**001
Supine SA	0**.**147	0**.**089	0**.**175	0**.**043
Standing SA	0**.**149	0**.**086	0**.**189	0**.**028
Supine LCEA	−0**.**615	0**.**000	−0**.**629	<0**.**001
Standing LCEA	−0**.**620	0**.**000	−0**.**688	<0**.**001
WEIGHT	−0**.**095	0**.**271	−0**.**121	0**.**161
BMI	−0**.**111	0**.**198	−0**.**117	0**.**175

**Table 3:** Correlation between TD changes and other hip parameters.

	TD Changes		TD Changes (TD > 10 mm)	
	Pearson Correlation	*P*-value	Pearson Correlation	*P*-value
Supine TA	0.100	0.247	0.130	0.288
Standing TA	0.104	0.231	0.096	0.435
Supine SA	−0.010	0.911	−0.045	0.716
Standing SA	−0.030	0.729	−0.094	0.444
Supine LCEA	−0.134	0.120	−0.122	0.317
Standing LCEA	−0.276	0.001	−0.313	0.009
WEIGHT	−0.079	0.362	−0.124	0.309
BMI	−0.045	0.608	−0.087	0.476

## Discussion

This study demonstrates that TD > 10 mm is a common imaging feature of DDH. DDH patients have an increased TD from the supine to the standing position. Lateral displacement of the center of femoral head can be used on evidence of hip instability for DDH. TD remains associated with TA and LCEA, respectively, which indicates that TD indirectly reflects the degree of hip dysplasia. Our results revealed a correlation between the TD difference and LCEA in the standing position, thus demonstrating that the worse lateral coverage of the femoral head was associated with a more unstable hip.

Hip stability depends on bony and soft tissue structure. Generally, the hip joint is considered a highly matched concentric ball and socket joint. However, several anatomic and finite element analysis studies had proved that the femoral head and acetabulum relationship is not actually a perfectly congruent ball and socket joint. A parallel displacement of the articular surface is achieved under a load, with the translation of up to 2–5 mm from the hip center (21, 22). Indeed, this conclusion, which is based on laboratory studies, is different from the physiological state of the muscles surrounding the hip joint in healthy populations.

The hip supports upper body weight in daily activities with a wide range of motion. Hip instability is generally defined as the manifestation that causes pain when the hip is physically moving, with or without hip instability (23). Many methods have been reported to reflect the relationship of the femoral head and acetabulum during exercise, but the subjects of these studies mainly focused on the normal population or patients with FAI (11–15). DDH is a common bony pathogenic factor leading to hip instability in adolescents and young adults, but few studies have reported the evaluation methods of hip instability in DDH patients.

To investigate the relationship between the femoral head and acetabular position, Siebenrock et al. proposed the shortest distance between the medial edge of the femoral head and the iliocostal line as the horizontal parameter and between the inferior edge of the femoral head and the inferior edge of the teardrop as the vertical paramete (24). Using the above parameter Troelsen et al. found no abnormal displacement of the femoral head in DDH patients during position changes (20). However, their results were limited due to the relatively small sample size. In addition, other studies have reported that the variation in the sagittal pelvic tilt between the supine and standing positions, and the vertical distance parameter was affected by the variation in the sagittal pelvic tilt, which is the main disadvantage of the vertical distance parameter for evaluating hip instability (25, 26).

Sweeney et al. (27) proposed the concept of the teardrop distance and defined it as the distance between the medial edge of the femoral head and the lateral edge of the teardrop in an anteroposterior radiograph of the pelvis, i.e., the shortest distance of the inner edge of the femoral head and the bottom of the acetabular fossa. TD more directly reflects the horizontal relationship between the femoral head and the bottom of the acetabular fossa. Theoretically, TD in different weight-bearing states should change with a displacement of the femoral head and acetabulum under hip instability conditions.

Our results showed that TD was significantly higher in the DDH group than in the normal group. Although not a diagnostic indicator, TD > 10 mm should be a common imaging manifestation in DDH. Clohisy et al. (24) reported that if the inner edge of the femoral head and iliocostal distance were >10 mm, extra central displacement of the hip joint was considered (28).

LCEA and TA are common parameters used in hip evaluation. The more severe the hip dysplasia, the smaller the LCEA and the larger the TA. Our results revealed the correlation of TD with LCEA and TA, thus indicating that TD could reflect the severity of DDH. There was a moderate correlation between TD and LCEA/TA, suggesting that TD could also reflect the severity of hip dysplasia. In cases with large TD, hip dysplasia was more serious, and this phenomenon was more obvious in cases with TD > 10 mm. The TD difference from supine to standing position in the DDH group was correlated with standing LCEA. Femoral head displacement was related to the degree of hip dysplasia. This correlation was more significant with TD > 10 mm. LCEA, TA, and TD are radiographic-specific parameters describing DDH that cannot be used as direct evidence of hip instability. We selected TD as a parameter of the hip joint and compared the anteroposterior radiographs of the pelvis at two positions to obtain the changing trend of the hip joint parameters under different loads as an initial assessment of hip instability in DDH patients. The hip is required to bear upper body weight when standing compared to the supine position. The hip instability can be considered if the femoral head displaced laterally from the fossa acetabuli under two different loading conditions. TD in the standing position was wider than that in supine position in the DDH group, thus indicating that female DDH patients had a horizontal external displacement of the femoral head during weight-bearing as the manifestation of hip instability. Although statistically TD changes were only 0.4 mm, we believed that reproducibility was needed to be prospectively validated by larger studies. The TD difference from supine to standing position in the DDH group was correlated with standing LCEA. Although the correlation was not strong, femoral head displacement was related to the degree of hip dysplasia. This correlation was more significant with TD > 10 mm.

True hip dysplasia is defined by a radiographic LCEA of approximately <20° and a borderline dysplasia between 20° to 25° (29). Borderline dysplasia of the hip is difficult to be recognized because of multiplanar hip instability caused by any of several patterns of joint deformity. Patients with borderline hip dysplasia present a challenging treatment dilemma, as it remains unknown when they should be treated with hip arthroscopy and/or a PAO (30). Although radiographic findings of borderline dysplasia might suggest instability, the ultimate diagnosis is based on history and physical exam, as well as imaging. The FEAR Index can present excellent reliability and improve the ability to distinguish unstable hips from stable hips with borderline dysplasia (31). But osseous deficiency can occur in any of the three developmental ossifications that contribute to stability of the hip, which are affected in a dynamic three planar interaction. We found that TD was really adding another measurement to quantify DDH, and TD changes from supine to standing indicated hip instability in DDH. Another important potential power of TD changes was associated with joint loading in the borderline dysplasia patients. If these patients were shown to have increased TD under stress, it would increase concern for unstable labral problems. As its utility increasing, more critical data will strengthen the scope of how we make treatment decisions. Combined with a thorough physical examination, stable versus unstable borderline dysplastic hips will receive the correct diagnosis and treatment plan.

There are some limitations in the present study. First, the sample size was too small to adequately indicate subtle differences between normal subjects and DDH patients. The mechanism and measurement of anteroposterior pelvic radiograph images may affect the accuracy of the result. The subjects of the study were female and did not apply to males, which lacked generalizability. Acetabular coverage is divided into anterior coverage, posterior coverage, and lateral coverage. However, the selected hip parameters in this study mainly reflected the lateral coverage of the acetabulum. TD should be measured as a routinely used parameter, but it is also affected by other factors, such as magnification error of radiographs and patient body size. Other factors such as the shape of the femoral head, congruency between acetabulum and femoral head, labrum injury or varus labrum, were not included in our study.

## Conclusion

TD > 10 mm was a common imaging feature of DDH. It increased from supine to standing position, thus indicating hip instability in DDH patients. The hip parameters of both positions should be compared, fully considering the factors of hip stability.

## Data Availability

The original contributions presented in the study are included in the article/Supplementary Material, further inquiries can be directed to the corresponding author/s.
